# Monoclonal gammopathy of renal significance (MGRS): Real‐world data on outcomes and prognostic factors

**DOI:** 10.1002/ajh.26566

**Published:** 2022-04-20

**Authors:** Alessandro Gozzetti, Andrea Guarnieri, Elena Zamagni, Elena Zakharova, Daniel Coriu, Max Bittrich, Tomáš Pika, Natalia Tovar, Natalia Schutz, Sara Ciofini, Camila Peña, Serena Rocchi, Michael Rassner, Irit Avivi, Anna Waszczuk‐Gajda, Saurabh Chhabra, Lidia Usnarska‐Zubkiewicz, Verónica González‐Calle, Maria‐Victoria Mateos, Monica Bocchia, Flavia Bigi, Hannah Füllgraf, Bhavna Bhasin‐Chhabra, Massimo Gentile, Julio Davila, David H. Vesole, Michele Cavo, Bicky Thapa, Edvan Crusoe, Hermann Einsele, Wojciech Legiec, Grzegorz Charliński, Artur Jurczyszyn

**Affiliations:** ^1^ Hematology, Department of Medical Science, Surgery and Neuroscience University of Siena Siena Italy; ^2^ Nephrology Azienda Ospedaliera Universitaria Senese Siena Italy; ^3^ IRCCS Azienda Ospedaliero‐Universitaria di Bologna Istituto di Ematologia “Seràgnoli” Bologna Italy; ^4^ Dipartimento di Medicina Specialistica Diagnostica e Sperimentale Università di Bologna Bologna Italy; ^5^ Department of Nephrology Moscow City Hospital named after S.P. Botkin Moscow Russian Federation; ^6^ Department of Hematology, Fundeni Clinical Institute University of Medicine and Pharmacy "Carol Davila" Bucharest Romania; ^7^ Department of Internal Medicine II University Hospital Würzburg Würzburg Germany; ^8^ Department of Hemato‐Oncology University Hospital Olomouc Olomouc Czech Republic; ^9^ Amyloidosis and Myeloma Unit, Department of Hematology, Hospital Clínic of Barcelona Institut d'Investigacions Biomèdiques August Pi i Sunyer (IDIBAPS), University of Barcelona Barcelona Spain; ^10^ Department of Hematology Hospital del Salvador Santiago Chile; ^11^ Faculty of Medicine, Department of Medicine I, Medical Center University of Freiburg Freiburg Germany; ^12^ Tel Aviv Medical Center, Tel Aviv, Israel and Sackler Faculty of Medicine Tel Aviv University Tel Aviv Israel; ^13^ Departament od Hematology, Transplantology and Internal Medicine Medical University of Warsaw Warsaw Poland; ^14^ Division of Hematology/Oncology, Department of Medicine Medical College of Wisconsin, Milwaukee Wisconsin USA; ^15^ Department of Hematology, Blood Neoplasms and Bone Marrow Transplantation Wroclaw Medical University Wroclaw Poland; ^16^ Instituto de Investigación Biomédica de Salamanca (IBSAL) University Hospital of Salamanca Salamanca Spain; ^17^ Department of Medicine Division of Nephrology, Medical College of Wisconsin Milwaukee Wisconsin USA; ^18^ Hematology Unit, Department of Onco‐Hematology Cosenza Italy; ^19^ Department of Hematology Complejo Asistencial de Avila Avila Spain; ^20^ The John Theurer Cancer Center at Hackensack Meridian School of Medicine Hackensack New Jersey USA; ^21^ Federal University of Bahia University Hospital, Rede D'or Oncology Sao Paolo Brazil; ^22^ Department of Hematology and Bone Marrow Transplantation St. John of Dukla Oncology Center of Lublin Land Lublin Poland; ^23^ Department of Hematology, Medical Faculty University of Warmia and Mazury in Olsztyn Olsztyn Poland; ^24^ Plasma Cell Dyscrasias Center, Department of Hematology Jagiellonian University Medical College, Faculty of Medicine Cracow Poland

## Abstract

Monoclonal gammopathy of renal significance (MGRS) is a recognized clinical entity. Literature regarding treatment and its outcomes in MGRS is sparse due to the rarity and misdiagnosis of MGRS. We retrospectively analyzed 280 adults with an MGRS diagnosis from 2003 to 2020 across 19 clinical centers from 12 countries. All cases required renal biopsy for the pathological diagnosis of MGRS. Amyloidosis‐related to MGRS (MGRS‐A) was present in 180 patients; nonamyloidosis MGRS (MGRS‐NA), including a broad spectrum of renal pathologies, was diagnosed in 100 patients. The median overall survival in the studied cohort was 121.0 months (95% CI: 105.0–121.0). Patients with MGRS‐A had a shorter overall survival than patients with MGRS‐NA (HR = 0.41, 95%CI: 0.25–0.69; *p* = 0.0007). Both hematologic and renal responses were associated with longer survival. Achievement of ≥VGPR was generally predictive of a renal response (OR = 8.03 95%CI: 4.04–115.96; *p* < 0.0001), one‐fourth of patients with ≥VGPR were renal nonresponders. In MGRS‐A, factors associated with poor prognosis included elevated levels of creatinine, beta‐2‐microglobulin, and hemodialysis at diagnosis. In MGRS‐NA, only age >65 years was associated with increased risk of death. Treatments provided similar hematologic response rates in both types of MGRS. Autologous stem cell transplantation led to better response than other treatments. This multicenter and international effort is currently the largest report on MGRS.

## 1 INTRODUCTION

Monoclonal gammopathy of undetermined significance (MGUS) is defined by a serum or urine monoclonal protein of less than 3 g/dL and 500 mg/24 h, respectively, and by less than 10% monoclonal plasma cells in the bone marrow.^1^ Currently, no treatment is indicated for MGUS outside of a clinical trial. The cumulative risk of progression to multiple myeloma (MM) has been widely reported and is about 1% per year in 20 years.^2^ Smoldering multiple myeloma (SMM) is characterized by 10%–60% monoclonal plasma cells in the bone marrow and/or ≥3 g/dL of monoclonal component in the serum or urine of ≥500 mg/24 h. In SMM, there is no organ damage, and treatment is indicated only in the context of a clinical trial.^3^, ^4^ Three new criteria have been introduced to predict increased risk of progression from SMM to symptomatic MM within two years from diagnosis: bone marrow plasma cells ≥60%, free light chain (FLC) ratio > 100 (or if κ/λ ratio is used, ≥100 or ≤0.01), and ≥1 bone lesion detected by magnetic resonance imaging (MRI)—now defined as SLiM‐CRAB.^3^ However, both MGUS and SMM may be harmful in the absence of the usual SLiM‐CRAB criteria. A newer term, monoclonal gammopathy of clinical significance (MGCS) indicates organ compromise secondary to the production of a clonal paraprotein(s), which damages the kidney, eye, heart, liver, or neurological compartment.^5^, ^6^, ^7^, ^8^, ^9^ In addition to MGUS and SMM, this may also be a manifestation of small B‐cell clones arising from lymphoproliferative diseases, that is, chronic lymphocytic leukemia (CLL) or non‐Hodgkin lymphoma (NHL).

In MGRS, a kidney damage is not due to light cast nephropathy.^10^, ^11^, ^12^, ^13^, ^14^ The mechanisms for organ damage are related to the physicochemical properties of the monoclonal immunoglobulin. A recent consensus statement for the diagnosis and treatment of MGRS was published by the International Kidney and Monoclonal Research Group (IKMG).^12^ MGRS is commonly underdiagnosed due to the rarity and lack of familiarity. The clonal deposition of monoclonal immunoglobulin does not respond to immunosuppression as in other nephropathies, tends to progress or relapse after kidney transplant, leading to end‐stage renal disease.^15^, ^16^, ^17^, ^18^, ^19^, ^20^, ^21^, ^22^ For all these reasons, the literature lacks the true incidence and optimal treatment of MGRS. The objective of this multi‐institutional study was to evaluate prognostic indicators and treatment outcomes in MGRS. Considering the high incidence of AL amyloidosis within MGRS, we compared outcomes of patients with amyloidosis and nonamyloidosis MGRS.

## 2 PATIENTS AND METHODS

This was a retrospective, international, multicenter study to survey the landscape of MGRS treatment and its outcomes in collaborating centers in Brazil, Chile, Czech Republic, Germany, France, Italy, Israel, Poland, Romania, Russia, Spain, and the United States. The individual institutional review boards approved the study. Personal data were deidentified to ensure compliance with relevant data privacy regulations. Data collection was based on standardized study forms and aggregation of information by the study coordinator. The diagnostic criteria was consistent with the consensus statement from the IKMG.^12^ All patients included in the analysis required local hematopathological confirmation by renal biopsy for one of the following pathological diagnoses: AL amyloidosis, monoclonal immunoglobulin deposition disease (MIDD), proliferative glomerulonephritis (GN) with monoclonal immunoglobulin deposition disease (PGNMID), monoclonal fibrillary GN, immunotactoid GN, cryoglobulinemic GN, light chain proximal tubulopathy (LCPT), crystal‐storing histiocytosis, C3 glomerulopathy with monoclonal gammopathy, and thrombotic microangiopathy.^22^, ^23^, ^24^, ^25^, ^26^, ^27^ Light‐chain cast nephropathy was excluded since it was considered a myeloma‐defining event.^6^ The analyzed parameters included: age at diagnosis, gender, heavy and light chain isotype, serum‐free light‐chain κ and λ, the percentage of clonal plasma cells in bone marrow, the presence of B‐cell lymphoproliferative disease, fluorescence in situ hybridization (FISH) for cytogenetic abnormalities [t(14;16), t(4;14), TP53 and/or del 17p] or as per local guidelines^28^ hemoglobin level, serum concentrations of calcium, albumin, beta‐2‐microglobulin, LDH, creatinine, estimated glomerular filtration rate (eGFR), hemodialysis required at diagnosis and the duration of hemodialysis, frontline treatments, responses (hematologic and renal response), progression‐free survival (PFS), and OS. Treatment outcomes were classified by the Uniform International Myeloma Working Group (IMWG) criteria.^29^ Hematologic response was defined as complete response (CR) if normalization of FLC was obtained when available. Otherwise, a disappearance of monoclonal protein at electrophoresis and with serum or urinary immunofixation or disappearance of the plasmacellular clone. Defining very good partial response (VGPR) and partial response (PR) required 90% or 50% monoclonal protein reduction, respectively.

Renal response was assessed as a reduction of >30% of 24 h proteinuria (in the absence of renal progression defined by progressive decrease of >25% of eGFR).^30^

Treatments were divided into proteasome inhibitor–based (bortezomib, carfilzomib or ixazomib, i.e., PI), immunomodulatory (IMiD) drug–based (thalidomide, lenalidomide, pomalidomide, i.e., IMiD), monoclonal antibody–based (daratumumab, rituximab, i.e., MoA), corticosteroids–prednisone, dexamethasone), chemotherapy‐based (chemotherapy alone), and autologous stem cell transplantation (ASCT).

### 2.1 Statistical analysis

The chi‐square test and the Mann–Whitney U‐test were used to compare categorical and continuous variables, respectively. For the survival analysis, the Kaplan–Meier method was used to generate survival curves, which were then compared using the log‐rank test. The Cox proportional‐hazard regression method was used to fit univariate and multivariate survival models, the results of which are reported as hazard ratios (HRs) with 95% confidence intervals (95% CIs). Variables with >50% of missing data were not included in the survival analyses. All reported *p*‐values are two‐sided and were considered significant if less than 0.05. All analyses were performed with RStudio Version 1.4.1106, and figures were prepared using MedCalc version 20.014 (MedCalc Software Ltd. Ostend, Belgium). Variables included in the univariate and multivariate models were age, gender, serum concentrations of creatine, albumin, beta‐2microglobulin, LDH, FLC κ/λ, and hemodialysis dependence. The hematologic response was analyzed as CR and VGPR, PR and stable disease (SD), and progressive diseases (PD).

All analyses were carried out in groups of patients with amyloidosis‐related MGRS (MGRS‐A) related and non‐amyloidosis‐related MGRS (MGRS‐NA).

## 3 RESULTS

### 3.1 Patients

From 331 patients initially reported, 51 were excluded from analysis due to the lack of clear MGRS diagnosis, that is, unconfirmed by renal biopsy or having symptomatic MM. From January 2003 to June 2020, 280 patients were diagnosed with MGRS. Only 9% of patients (*n* = 26) were diagnosed before 2010. Patient characteristics are reported in Table 1. Two‐thirds of patients had MGRS‐A (64%). Over half of patients with MGRS‐NA had MIDD. More patients in the MGRS‐NA group had IgG disease and serum‐free light chain κ compared with patients in the MGRS‐A group (Table 1). Renal impairment was more severe in patients with MGRS‐NA than in the group with MGRS‐A (Table 1). MGRS‐associated clonal diagnoses were MGUS (*n* = 214, 76.4%), SMM (*n* = 55, 19.6%) and non‐Hodgkin lymphoma (*n* = 9, 5.0%, including 6 patients with Waldenström's macroglobulinemia, 2 with lymphoplasmacytic lymphoma and 1 with marginal zone lymphoma). FISH was available for 76/280 patients, and t(11;14) was present in 13 patients, gain 1q21 in 5 patients, del17p in 1 patient.

**TABLE 1:** Patient's characteristics

	MGRS‐A	MGRS‐NA	*p*‐value
Number of patients	180	100	
Median age (range), years	61 (28–87)	60 (25–87)	0.2790
Male sex, *n* (%)	90 (50.0%)	51 (49.0%)	0.8728
Monoclonal component			
Heavy chains, *n* (%)			<0.0001
IgA κ/ λ	1/9 (5.8%)	3/3 (6.2%)	
IgG κ/ λ	10/41 (29.7%)	39/16 (56.7%)	
IgM κ/ λ	4/8 (7.0%)	5/1 (6.2%)	
Free light chains			
κ	24 (14.0%)	23 (23.7%)	
λ	75 (43.6%%)	7 (7.2%)	
Type of MGRS			
MIDD	Not applicable	53 (53%)	‐
PGNMID		14 (14%)	
LCPT		11 (11%)	
Monoclonal fibrillary GN		4 (4%)	
Immunotactoid GN		4 (4%)	
C3 glomerulopathy with monoclonal gammopathy		7 (7%)	
Other		5 (5%)	
Cryoglobulinemic GN		2 (2%)	
Laboratory parameters, median (range)			
Bone marrow involvement (% PCs), median* (range)	5.7% (0–50)	7.5% (0–55)	0.1386
Monoclonal component (mg/dL), median* (range)	5.0 (0–3000)	57 (0–2460)	0.0763
FLC κ, median (range) (mg/dL), median* (range)	21.1 (0–2152)	71.4 (1.4–6680)	<0.0001
FLC λ, (mg/dL), median* (range)	21.8 (0–4260)	68.6 (0–1815)	<0.0001
FLC κ/λ, median* (range)	0.4 (0–309)	3.5 (0–1040)	< 0.0001
Albumin (≥3.5 mg/dL), *n* (%)	153 (93.3%)	85 (92.4%)	0.7871
B‐2‐microglobulin (≥5.5 mg/L), *n* (%)	26 (34.2%)	34 (54.8%)	0.0055
LDH ≥300 U/L, *n* (%)	46 (36.8%)	22 (26.5%)	0.1221
Creatinine ≥177 mg/dL, *n* (%)	51 (32.3%)	58 (58.0%)	<0.0001
eGFR <60 ml/min/1.73m^2^, *n* (%)	97 (54.5%)	72 (88.8%)	<0.0001
24 h urine protein (g)	6.2 (0.1–14 850)	3.4 (0–7000)	0.0001
Dialysis, *n* (%)	33 (18.3%)	26 (26.0%)	0.0004
Treatment, *n* (%)			
Untreated	25 (13.9%)	12 (12.0%)	0.0614
1 line	106 (58.9%)	65 (65.0%)	
2 lines	35 (19.4%)	17 (17.0%)	
3 lines	14 (7.8%)	6 (6.0%)	

Abbreviations: eGFR, estimated glomerular filtration rate; FLC, free light chains; GN, glomerulonephritis; LCPT, light chain proximal tubulopathy; LDH, lactate dehydrogenase; MGRS‐A, amyloidoid‐associated monoclonal gammaglobulinemia of renal significance; MGRS‐NA, non‐amyloidosis‐associated gammaglobulinemia of renal significance; MIDD, monoclonal immunoglobulin deposition disease; PGNMID, proliferative glomerulonephritis with monoclonal immunoglobulin deposition disease monoclonal fibrillary glomerulonephritis.

* Values based on the nonmissing data.

Typically, untreated patients refused treatment or were referred and lost to follow‐up (20/37, 51.3%) or died within two years from the diagnosis (9/37, 24.3%). Causes of death included multiorgan failures (*n* = 3), disease progression (*n* = 3), sepsis (*n* = 2), and gastrointestinal bleeding (*n* = 1). Response and survival analyses were performed only among patients who received treatment.

### 3.2 Treatment and response

The majority of patients received treatment (87%). Frontline treatments and the corresponding hematologic responses are summarized in Table S1. The overall response rate (ORR) was 56% and 72% in the MGRS‐A and MGRS‐NA groups, respectively. The most common first‐line treatment was PI‐based followed by conventional chemotherapy. Overall, 16% of patients received ASCT as part of the first‐line treatment. There were no differences in hematologic responses regardless of the induction regimens and/or ASCT in patients with different types of MGRS. There was no difference in the rate of ≥VGPR between patients with MGRS‐A (37%, 45/122) and MGRS‐NA (43%, 34/78), *p* = 0.3449. Around 30% of patients received the second line of treatment, and <10% also the third line. The rate of ≥VGPR in the subsequent lines of treatment was increasing (Table S2).

Both hematologic and renal responses were available in 74% of treated patients (182/243). Among patients with ≥VGPR, 73.8% (31/42) and 76.9% (20/26) achieved renal response in the MGRS‐A and MGRS‐NA groups, respectively. A renal response was less common than hematologic. Overall, in patients with ≥VGPR, 25% did not achieve a renal response. Among patients with kidney function evaluated, the renal response was confirmed in 39% of patients with MGRS‐A (50/129) and 60% of patients with MGRS‐NA (39/65), *p* = 0.0052. In the MGRS‐A group, among patients with both hematologic and renal response evaluated, the renal response was present in 73.8% (31/42) and 19.0% (12/63) of patients with ≥VGPR and PR or SD, respectively (*p* < 0.0001). In the MGRS‐NA group, the renal response was present in 76.9% (20/26) and 47.0% (16/34) of patients with ≥VGPR and PR or SD, respectively (*p* = 0.0202). There was no difference in the renal response between patients with ≥VGPR in both groups (*p* = 0.7759); however, patients with PR or SD in the MGRS‐NA group were more likely renal responders than in the MGRS‐A group (*p* = 0.0038).

In general, achievement of ≥VGPR increased the likelihood for a renal response (OR = 8.03 95%CI: 4.04–115.96; *p* < 0.0001).

### 3.3 Survival analysis and prognostic factors of survival

**FIGURE 1:**
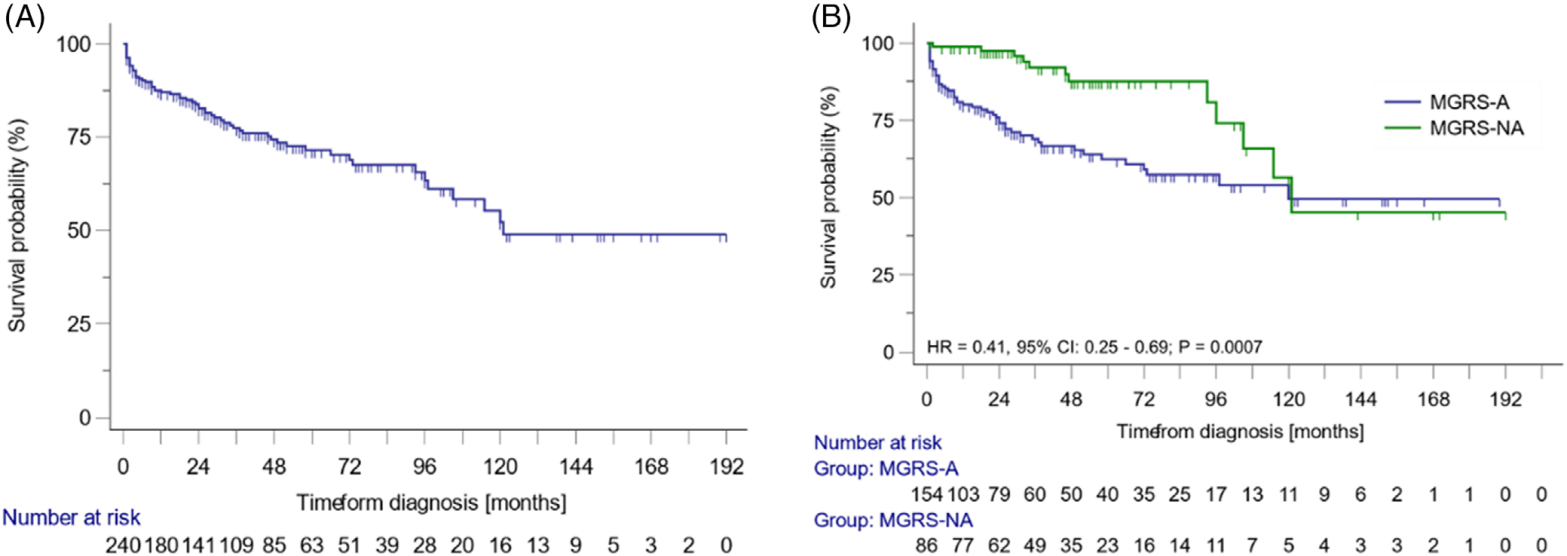
Overall survival of all patients and amyloidosis‐associated monoclonal gammopathy of renal significance (MGRS‐A) and non‐amyloidosis‐associated gammopathy of renal significance (MGRS‐NA). Overall survival of all patients with monoclonal gammopathy of renal significance (A) and overall survival of patients with MGRS‐A, and MGRS‐NA (B) [Color figure can be viewed at wileyonlinelibrary.com]

Survival analysis was performed only among patients who received treatment. After a median follow‐up of 30 months (range 1–192 months), the median OS was 121.0 months (95%CI: 105.0–121.0) (Figure 1A, B), Patients with MGRS‐NA were at a significantly lower risk of death than patients with MGRS‐A (HR = 0.41, 95% CI: 0.25–0.69; *p* = 0.0007). This effect was observed during the first 120 months, and the curves converged since that time, with 50% of patients in both groups remaining alive. There were more deaths in the MGRS‐A (31%, 51/154) compared to the MGRS‐NA group (14%, 12/86); *p* = 0.0130. The most common causes of death in the MGRS‐A group included disease progression (29%), infection (20%), and heart failure (16%); in 20% of cases, a cause of death was not recorded. In the MGRS‐NA group, disease progression (42%) and infections (25%) were the most common causes of death. In contrast to the MGRS‐A group, only one case of death in the MGRS‐NA group was related to a cardiovascular event. Thirty‐seven patients either refused treatment or were referred and lost to follow‐up (20/37, 51%) or died within two years from the diagnosis (9/37, 24%). Causes of death included multiorgan failure (*n* = 3), disease progression (*n* = 3), sepsis (*n* = 2), and gastrointestinal bleeding (*n* = 1).

**FIGURE 2:**
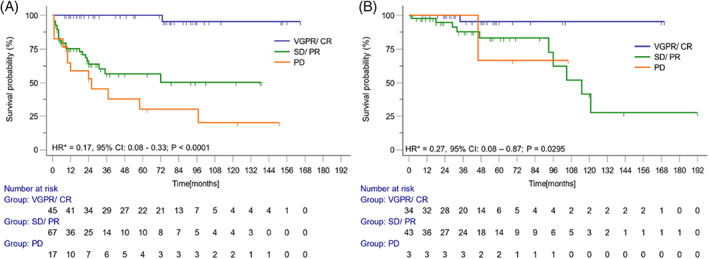
Overall survival and hematological response. Overall survival in the groups with amyloidosis‐associated monoclonal gammopathy of renal significance (A) and non‐amyloidosis‐associated gammopathy of renal significance (B) depending on hematological response.* ‐ Hazard ratio for comparison between survival of patients with ≥VGPR versus ≤PR or PD [Color figure can be viewed at wileyonlinelibrary.com]

**FIGURE 3:**
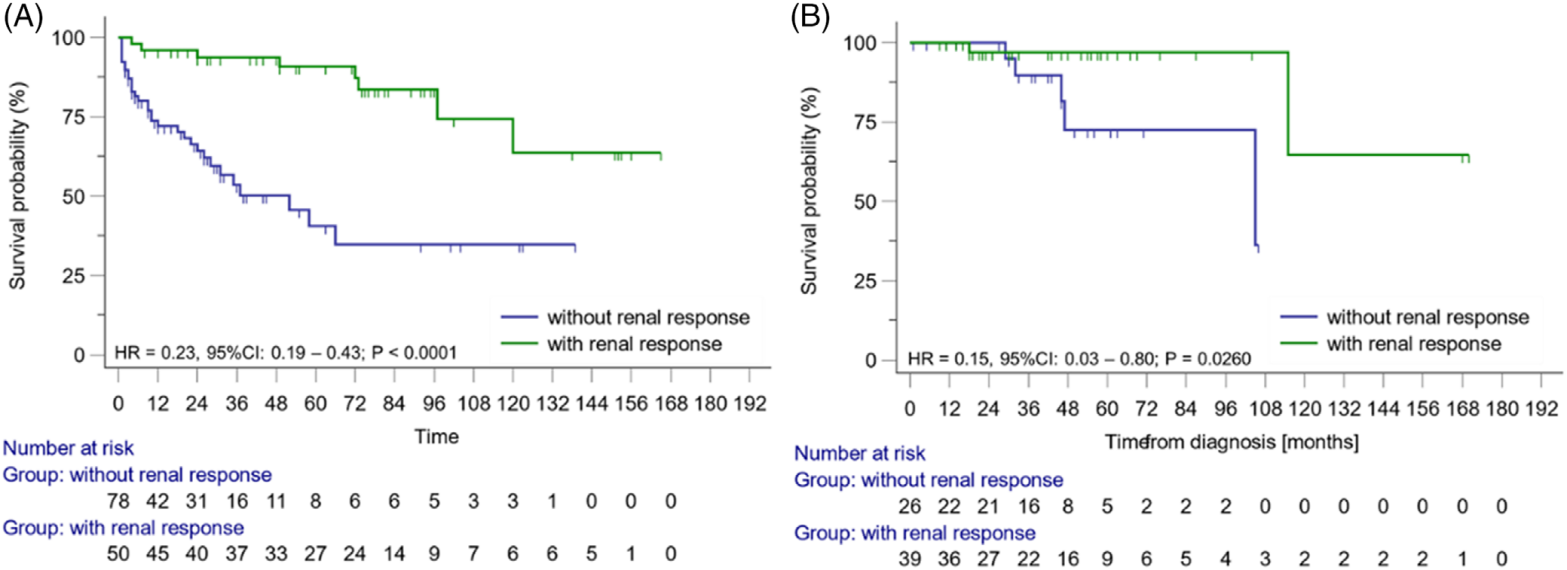
Overall survival and renal response. Overall survival in the groups with amyloidosis‐associated monoclonal gammopathy of renal significance (A) and non‐amyloidosis‐associated gammopathy of renal significance (B) depending on renal response [Color figure can be viewed at wileyonlinelibrary.com]

Achievement of ≥VGPR after the first‐line of treatment was associated with improved survival in the MGRS‐A group (HR = 0.17, 95% CI: 0.08–0.33; *p* < 0.0001) and in the MGRS‐NA group (HR = 0.27, 95% CI: 0.08–0.87; *p* = 0.0295) (Figure 2). The renal response was associated with better survival both in the MGRS‐A (HR = 0.23, 95%CI: 0.19–0.43; *p* < 0.0001) and MGRS‐NA group (HR = 0.15, 95%CI: 0.03–0.80; *p* = 0.0260) (Figure 3). In a univariate analysis, elevations of beta‐2‐microglobulin and creatinine, as well as hemodialysis dependence, were associated with increased risk of death in the MGRS‐A group (Table S3). None of these factors were significant in the multivariate analysis (beta‐2‐microglobulin HR = 1.66, 95% CI: 0.52–5.28, *p* = 0.556; creatinine HR = 1.41, 95% CI: 0.45–4.43; *p* = 0.556; hemodialysis HR = 1.87, 95% CI: 0.72–4.85, *p* = 0.201). For the MGRS‐NA, patients >65 years old had an increased risk of death compared to the younger group.

## 4 DISCUSSION

This is the largest report of biopsy‐proven MGRS.^16^, ^17^, ^18^, ^19^, ^20^, ^21^, ^22^, ^23^, ^24^, ^25^ The results comprise the real clinical practice of MGRS management, mainly in the past decade. Of note, many of these patients did not meet the criteria for myeloma, so only a subset received PIs or IMiDs as would be utilized in myeloma.

Patients with MGRS are heterogeneous. Different types of paraprotein‐associated renal involvement include all elements of the nephron.^12^, ^31^, ^32^ Primary differentiation between the most common MGRS‐A and MGRS‐NA revealed that these entities differed in disease burden and survival. Renal disease was more severe in patients with MGRS‐NA than with MGRS‐A, with a higher proportion of patients with elevated creatinine, low eGFR, and more frequently requiring hemodialysis in MGRS‐NA than in MGRS‐A. Despite similar bone marrow plasma cell percentages in MGRS‐NA and MGRS‐A, M‐protein levels were higher in MGRS‐NA. In addition, κ light chain paraprotein was more frequently produced in MGRS‐NA than in MGRS‐A, whereas FLC κ/λ was lower in MGRS‐A than in MGRS‐NA.^27^

Despite a lower clonal and renal burden, patients with MGRS‐A had shorter OS than patients with MGRS‐NA. Univariate analysis revealed that elevated creatinine level, requiring hemodialysis at diagnosis, and elevated beta‐2‐microglobulin were associated with a higher risk of death in the MGRS‐A group. None of these was an independent prognostic factor in multivariate analysis. However, there were no differences between groups in the hematologic response; patients with MGRS‐A had the renal response less likely than in the MGRS‐NA group. The second most frequently involved organ in AL amyloidosis is the heart. Cardiac involvement is the key driver of disease prognosis and mortality.^33^, ^34^, ^35^, ^36^, ^37^ We observed more deaths due to heart failure in the MGRS‐A group than in the MGRS‐NA group. These findings are similar to AL amyloidosis, in which mortality rates depend on cardiac dysfunction rather than the renal function.^35^ The above indirect evidence and the literature review support the importance of cardiac‐related mortality in MGRS‐A. Based on the study results, we cannot definitively explain excess mortality observed in MGRS‐A. However, in MGRS‐A 8/51 (16%), deaths were related to cardiac failure, while in MGRS‐NA only 1/12 (1%) death was related to cardiac failure, and we can assume that cardiac involvement was a possible prognosticator. The contribution of other factors can not be excluded. For example, the dosing and timing of dexamethasone use in AL amyloidosis with cardiac involvement have been associated with early mortality.^37^

Despite confirmation of the diagnosis with a renal biopsy, approximately 10% of the patient were not treated. In the case of MGRS, no treatment or delayed treatment are factors associated with a poor prognosis.^16^, ^17^

Management of MGRS requires monitoring by a hematologist and nephrologist.^7^ This remains an unmet medical need since 25% of patients had not assessed hematologic and/or renal response.^8^ Treatment of MGRS requires targeting of the underlying plasma cell or lymphoplasmacytic clone. Antimyeloma agents, typically bortezomib‐based regimens, were commonly used in plasmacytic disorders and anti‐CD20 immunotherapy in cases related to B‐cell lymphoproliferative disorders.^38^, ^39^ Alkylating agents were commonly used in our study population, whereas other antimyeloma drugs like PIs, IMiDs, and monoclonal antibodies were rarely used due to lack of availability in many countries at the analyzed time of the study. The majority of treated patients received only one line of treatment. In our study, different frontline regimens had similar efficacy in both types of MGRS. ASCT, in the frontline treatment, was the only treatment with a higher ORR.^40^, ^41^ Hematologic response increased chances for renal response and was associated with better survival. However, one‐fourth of patients did not improve renal function despite achieving ≥VGPR. This makes MGRS an entity with a significant medical need.

The study has some important limitations associated with its retrospective nature and limited follow‐up information. A limitation was the lack of information about the involvement of other organs than the kidney.

## 5 CONCLUSIONS

MGRS is a recognized and heterogeneous entity that requires treatment upon diagnosis. A significant proportion of patients remain untreated or not adequately followed up by hematologists and nephrologists. Typically, MGRS‐NA has a higher clonal and renal burden than MGRS‐A. MGRS‐A was associated with shorter survival, even though the biochemical disease burden was lower than in MGRS‐NA. Although direct evidence is not available, MGRS‐A probably has higher mortality due to cardiac involvement. The exact reason of higher mortality in MGRS‐A needs to be further investigated. Heart failure, contributing to 16% of reasons of death in MGRS‐A, cannot itself explain the difference in survival between two types of MGRS. Treatment and outcomes were similar in different types of MGRS. Whether novel treatments such as anti‐CD38 monoclonal antibodies will change responses and survival requires further investigations.

## CONFLICT OF INTEREST

All authors declare that they have no conflicts of interest.

## Supporting information


**Table S1.** First‐line treatment and hematologic response in patients with amyloidosis‐associated and non‐amyloidosis‐associated monoclonal gammopathy of renal significance.
**Table S2.** Patients with very good partial response of better in the subsequent lines of treatment. Data presented only of treated patients with evaluation of the response.
**Table S3.** Baseline patients characteristics with univariate analysis.

## Data Availability

The data that support the findings of this study are available on request from the corresponding author [Alessandro Gozzetti].
