# Supplementary material for: Monoclonal gammopathy of renal significance (MGRS): Real‐world data on outcomes and prognostic factors
Source: Am J Hematol. 2022 Apr 20;97(7):877–84. doi: 10.1002/ajh.26566 (PMC9324084; doi:10.1002/ajh.26566)
Supplement: Supplementary file 1 — Table S1. First‐line treatment and hematologic response in patients with amyloidosis‐associated and non‐amyloidosis‐associated monoclonal gammopathy of renal significance. Table S2. Patients with very good partial response of better in the subsequent lines of treatment. Data presented only of treated patients with evaluation of the response. Table S3. Baseline patients characteristics with univariate analysis. [file AJH-97-877-s001.docx]

**Supplementary Material.**

**Table 2. First-line treatment and hematologic response in patients with amyloidosis-associated and non-amyloidosis-associated monoclonal gammopathy of renal significance.**

| Treatment | Patient group (total number of patients treated) | Number evaluated | CR/ VGPR, n (%) | PR/ SD, n (%) | PD, n (%) | *P*-value |
| --- | --- | --- | --- | --- | --- | --- |
| All treated | MGRS-A (n = 155) | 130 | 45 (34.6) | 68 (52.3) | 17 (13.1) | 0.158 |
|  | MGRS-NA (n= 88) | 80 | 34 (42.5) | 43 (53.8) | 3 (3.8) |  |
| PI-based | MGRS-A (n = 101) | 81 | 30 (37.0) | 41 (50.6) | 10 (12.3) | 0.228 |
|  | MGRS-NA (n = 57) | 53 | 20 (37.7) | 33 (62.3) | 0 |  |
| Chemotherapy | MGRS-A (n = 38) | 41 | 13 (31.7) | 24 (58.5) | 4 (9.8) | 0.163 |
|  | MGRS-NA (n = 17) | 10 | 4 (40.0) | 4 (40.0) | 2 (20.) |  |
| IMiD-based | MGRS-A (n = 5) | 5 | 1 (20.0) | 3 (60.0) | 1 (20.0) | NE |
|  | MGRS -NA (n = 6) | 4 | 3 (75.0) | 1 (25.0) | 0 |  |
| PI+IMiD | MGRS-A (n = 1) | 1 | 1 (100) | 0 | 0 | NE |
|  | MGRS-NA (n = 7) | 3 | 1 (33.3) | 2 (66.7) | 0 |  |
| Steroid | MGRS-A (n = 2) | 2 | 0 | 0 | 2 (100) | NE |
|  | MGRS-NA (n =5) | 4 | 2 (50.0) | 2 (50.0) | 0 |  |
| IMiD + mAb | MGRS-A (n = 2) | 0 | 0 | 0 | 0 | NE |
|  | MGRS-NA (n = 2) | 3 | 1 (33.3) | 2 (66.7) | 0 |  |
| + ASCT | MGRS-A (n = 18) | 24 | 19 (79.2) | 4 (16.7) | 1 (4.2) | 0.158 |
|  | MGRS -NA (n = 26) | 16 | 12 (75.0) | 4 (25.0) | 0 |  |

ASCT, autologous stem cell transplantation; CR, complete response; mAb, monoclonal antibody; IMiD, immunomodulatory drug; MGRS-A, amyloidosis-associated monoclonal gammaglobulinemia of renal significance; MGRS-NA, non-amyloidosis-associated gammaglobulinemia of renal significance; PD, progressive disease; PI, proteasome inhibitor; PR, partial response; SD, stable disease; VGPR, very good partial response.

**Table 3.** Patients with very good partial response of better in the subsequent lines of treatment. Data presented only of treated patients with evaluation of the response.

|  | **1^st^ Line** | **2^nd^ line** | **3^rd^ line** |
| --- | --- | --- | --- |
| MGRS-A, patients with ≥VGPR, % (n/N) | 34.6% (45/130) | 26.8% (11/41) | 41.7% (5/12) |
| MGRS-NA, patients with ≥VGPR, % (n/N) | 42.5% (34/80) | 50.0% (11/22) | 83.3% (5/6) |

MGRS-A, amyloidosis-associated monoclonal gammaglobulinemia of renal significance; MGRS-NA, non-amyloidosis-associated gammaglobulinemia of renal significance; VGPR, very good partial response.

**Table 4. Baseline Patients Characteristics with univariate analysis**

| **Independent variables** | **Patient group** | **Level** | **n (%)** | **OS  (mean)** | **HR** | **95% CI** | **P- value** |
| --- | --- | --- | --- | --- | --- | --- | --- |
| Age, years | MGRS-A | ≤ 65 | 105 (67.7%) | 40.75 | 1.60 | 0.91 - 2.79 | 0.102 |
|  |  | > 65 | 50 (32.3%) | 41.82 |  |  |  |
|  | MGRS-NA | ≤ 65 | 62 (72.1%) | 51.55 | 3.85 | 1.14 - 13.0 | **0.029** |
|  |  | > 65 | 24 (27.9%) | 48.29 |  |  |  |
| Sex | MGRS-A | Male | 78 (50.3%) | 40.40 | 0.69 | 0.39 - 1.20 | 0.191 |
|  |  | Female | 77 (49.7%) | 41.78 |  |  |  |
|  | MGRS-NA | Male | 43 (50.0%) | 43.35 | 2.85 | 0.79 - 10.25 | 0.108 |
|  |  | Female | 43 (50.0%) | 57.93 |  |  |  |
| LDH (U/L) | MGRS-A | < 300 | 72 (46.5%) | 39.79 | 1.41 | 0.74 - 2.70 | 0.299 |
|  |  | ≥ 300 | 43 (27.7%) | 51.42 |  |  |  |
|  | MGRS-NA | < 300 | 56 (65.1%) | 45.80 | 1.35 | 0.39 - 4.67 | 0.638 |
|  |  | ≥ 300 | 19 (22.1%) | 53.10 |  |  |  |
| FLC κ/λ | MGRS-A | Normal | 27 (17.4%) | 42.96 | 1.345 | 0.64 - 2.80 | 0.435 |
|  |  | Abnormal | 83 (53.5%) | 51.17 |  |  |  |
|  | MGRS-NA | Normal | 16 (18.6%) | 48.69 | 1.53 | 0.29 - 7.94 | 0.615 |
|  |  | Abnormal | 57 (66.3%) | 52.88 |  |  |  |
| Albumin (mg/dL) | MGRS-A | < 3.5 | 10 (6.5%) | 86.66 | 4.86 | 0.66 - 35.58 | 0.120 |
|  |  | ≥ 3.5 | 131 (84.5%) | 37.50 |  |  |  |
|  | MGRS-NA | < 3.5 | 7 (8.1%) | 57.00 | 0.84 | 0.10 - 6.97 | 0.870 |
|  |  | ≥ 3.5 | 72 (83.7%) | 48.07 |  |  |  |
| Beta-2-microglobulin  (mg/L) | MGRS-A | < 5.5 | 50 (32.3%) | 45.38 | 2.52 | 1.08 - 5.87 | **0.031** |
|  |  | ≥ 5.5 | 24 (15.5%) | 35.08 |  |  |  |
|  | MGRS-NA | < 5.5 | 27 (31.4%) | 42.70 | 1.61 | 0.31 - 8.44 | 0.575 |
|  |  | ≥ 5.5 | 29 (33.7%) | 58.34 |  |  |  |
| Creatinine  (mg/dL) | MGRS-A | < 177 | 111 (71.6%) | 45.08 | 2.35 | 1.34 - 4.11 | **0.003** |
|  |  | ≥ 177 | 43 (27.7%) | 30.65 |  |  |  |
|  | MGRS-NA | < 177 | 38 (44.2%) | 43.13 | 0.98 | 0.30 - 3.18 | >0.9 |
|  |  | ≥ 177 | 48 (55.8%) | 56.58 |  |  |  |
| Dialysis | MGRS-A | Yes | 30 (19.4%) | 45.21 | 1.85 | 1.03 - 3.35 | **0.041** |
|  |  | No | 125 (80.6%) | 40.14 |  |  |  |
|  | MGRS-NA | Yes | 26 (30.2%) | 57.42 | 0.94 | 0.28 - 3.13 | >0.9 |
|  |  | No | 60 (69.8%) | 47.70 |  |  |  |

FLC, free light chain; LDH, lactate dehydrogenase; MGRS-A, amyloidosis-associated monoclonal gammaglobulinemia of renal significance; MGRS-NA, non-amyloidosis-associated gammaglobulinemia of renal significance.
